# The ETTAA study protocol: a UK-wide observational study of ‘Effective Treatments for Thoracic Aortic Aneurysm’

**DOI:** 10.1136/bmjopen-2015-008147

**Published:** 2015-06-02

**Authors:** Priya Sastry, Victoria Hughes, Paul Hayes, Srinivasa Vallabhaneni, Linda Sharples, Matt Thompson, Pedro Catarino, Narain Moorjani, Luke Vale, Joanne Gray, Andrew Cook, John A Elefteriades, Stephen R Large

**Affiliations:** 1 Papworth Hospital, Cambridge, UK; 2 Cambridge University Hospitals NHS Foundation Trust, Cambridge, UK; 3 Royal Liverpool Hospital, Liverpool, Merseyside, UK; 4 Clinical Trials Research Unit, University of Leeds, Leeds, UK; 5 St George's Hospital, London, UK; 6 Institute of Health & Society, Newcastle University, Newcastle upon Tyne, UK; 7 Southampton Health Technology Assessments Centre (SHTAC), University of Southampton, Southampton, Hampshire, UK; 8 Yale Center for Thoracic Aortic Disease, New Haven, Connecticut, USA

**Keywords:** VASCULAR MEDICINE

## Abstract

**Introduction:**

Chronic thoracic aortic aneurysm (CTAA) affecting the arch or descending aorta is an indolent but life-threatening condition with a rising prevalence as the UK population ages. Treatment may be in the form of open surgical repair (OSR) surgery, endovascular stent grafting (ESG) or best medical therapy (BMT). Currently, there is no consensus on the best management strategy, and no UK-specific economic studies that assess outcomes beyond the chosen procedure, but this is required in the context of greater demand for treatment and limited National Health Service (NHS) resources.

**Methods and analysis:**

This is a prospective, multicentre observational study with statistical and economic modelling of patients with CTAA affecting the arch or descending aorta. We aim to gain an understanding of how treatments are currently chosen, and to determine the clinical effectiveness and cost-effectiveness of the three available treatment strategies (BMT, ESG and OSR). This will be achieved by: (1) following consecutive patients who are referred to the teams collaborating in this proposal and collecting data regarding quality of life (QoL), medical events and hospital stays over a maximum of 5 years; (2) statistical analysis of the comparative effectiveness of the three treatments; and (3) economic modelling of the comparative cost-effectiveness of the three treatments. Primary study outcomes are: aneurysm growth, QoL, freedom from reintervention, freedom from death or permanent neurological injury, incremental cost per quality-adjusted life year gained.

**Ethics and dissemination:**

The study will generate an evidence base to guide patients and clinicians to determine the indications and timing of treatment, as well as informing healthcare decision-makers about which treatments the NHS should provide. The study has achieved ethical approval and will be disseminated primarily in the form of a Health Technology Assessment monograph at its completion.

**Trial registration number:**

ISRCTN04044627.

**Strengths and limitations of this study**

- As an observational study, we anticipate strong recruitment and a study that truly reflects UK practice.
- Since the study observed patients through the watchful waiting period into their treatment and beyond, we will be able to capture the elements that contribute towards decision-making.
- The planned Delphi exercise will deliver a consensus on which patients would be best served by which treatments.
- Being a UK-wide study, we will also be able to report on geographic variation in treatment availability and resource use.
- The planned health economic exercise is a particular strength of the study and will deliver a comprehensive cost-effectiveness analysis of the treatments available for chronic thoracic aortic aneurysm.
- Patient pathways may not be as clearly delineated as in a trial setting. We anticipate, therefore, that the interpretation of outcomes by group will need careful analysis. Sophisticated statistical modelling methods and a Delphi consensus exercise are planned to allow for this.

## Introduction

### The clinical problem

Chronic thoracic aortic aneurysm (CTAA) is defined by dilation of the thoracic aorta to beyond 50% of normal. Dilation weakens the aortic wall leading to aortic rupture, dissection and/or death. CTAA also increases the risk of non-fatal complications such as stroke, renal failure or paraplegia. There are estimated to be 3000–8000 new cases of CTAA in the UK each year.^1^ The natural history of CTAA is not clearly understood, however, because patients usually remain asymptomatic until presentation with rupture, dissection or death. CTAA can be detected prior to such events on imaging scans performed for other reasons. In these scenarios, there is a lack of evidence regarding what may happen without procedural intervention.

The rate of growth of CTAA has been estimated at 0.1 cm/year from Professor Elefteriades’s Yale database of 3000 patients with CTAA. His research demonstrated that aneurysm size (when indexed to the patient's size) is proportional to the risk of rupture (table 1).^2^ While the aneurysm is small, clinicians usually advise a period of watchful waiting (WW) during which the patient will have serial CT scans or MRI to monitor the size of the aneurysm. Medications will be started to control blood pressure and reduce the risk of rupture, dissection or death. Nonetheless, these (and other non-fatal) complications may still occur when the aneurysm is small. The risk factors for this are not clearly understood. The data we do have on the natural history of small aneurysms are retrospective. As such, it is likely to underestimate the risk of larger aneurysms (where patients are followed up for very short periods of time) and overestimate the risk of smaller aneurysms (where patients may be followed up for years). Therefore, a prospective study is required to describe the natural history of CTAA in asymptomatic patients prior to intervention.

**Table 1:** Risk of rupture according to indexed aortic size

Indexed aortic size, cm/m^2^	Risk of rupture, dissection or death, % per year
<2.75	4 (low risk)
2.75–4.25	8 (moderate risk)
>4.25	20 (high risk)

As aneurysms grow, there is an increasing risk of fatal complications. Treatment to reduce this risk can be in the form of best medical therapy (BMT), endovascular stent grafting (ESG) or open surgical repair (OSR), and is justified when the risk associated with intervention is perceived to be less than the risk of rupture, dissection or death. The risk of rupture is mostly (but not entirely) dependent on aneurysm size.^2^ The risk of intervention, however, is influenced by a variety of factors and is different for every patient. Therefore, specialist multidisciplinary teams play a key role in discussing the risk-benefit profile for each patient before recommending a treatment pathway. Ultimately, the choice of treatment is made by the patient after appropriate explanation and discussion.

**Figure 1:**
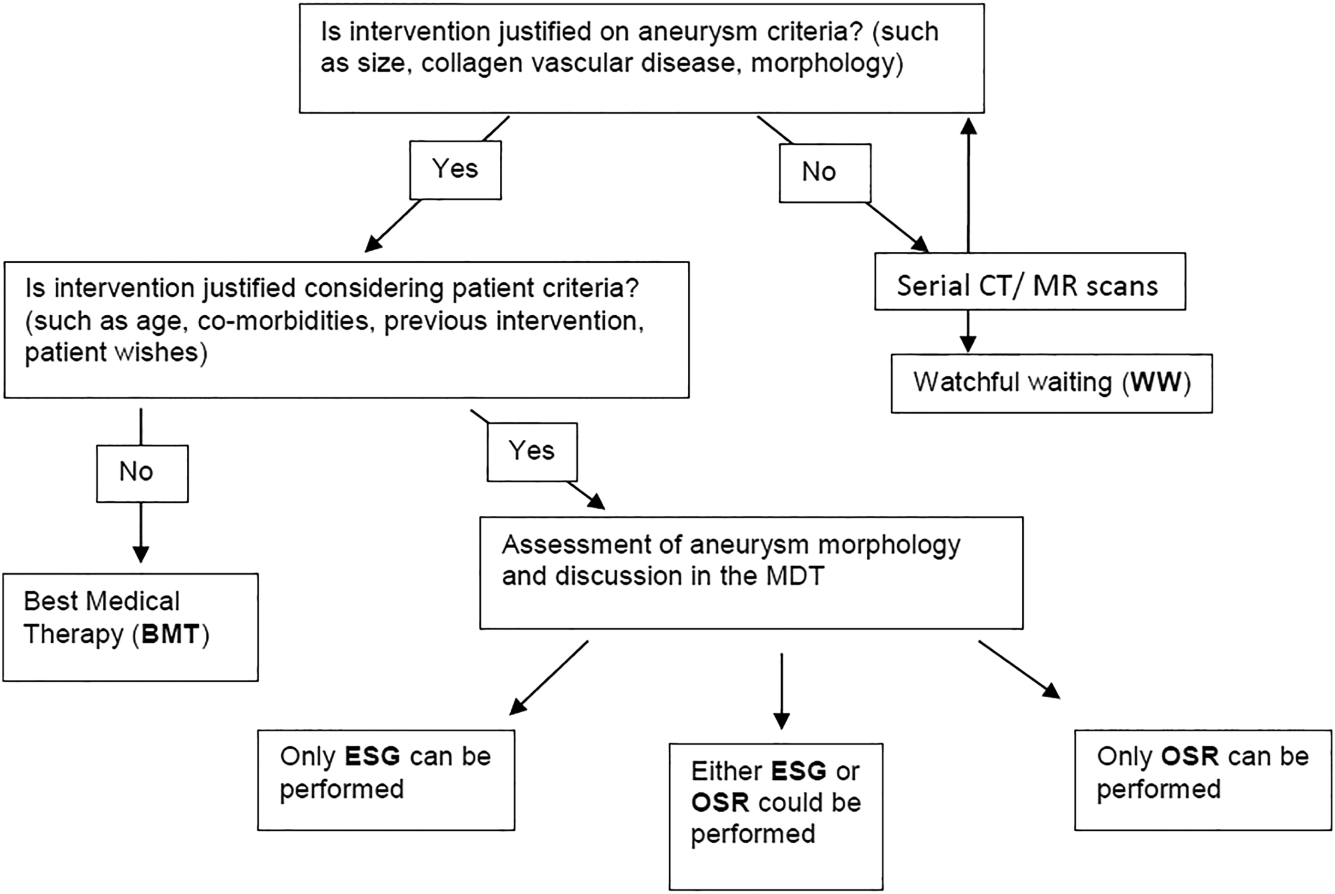
The decision-making process for treatment for chronic thoracic aortic aneurysm (CTAA; ESG, endovascular stent grafting; OSR, open surgical repair).

At present, there is no national or international guideline addressing patient selection for BMT, ESG or OSR. In the absence of such a guideline, the decision (BMT, ESG or OSR) is made after considering patient factors (including age, comorbidities and personal choice) and aneurysm factors (including indexed size, collagen vascular disorder and anatomy/morphology) (figure 1).

**Figure 2:**
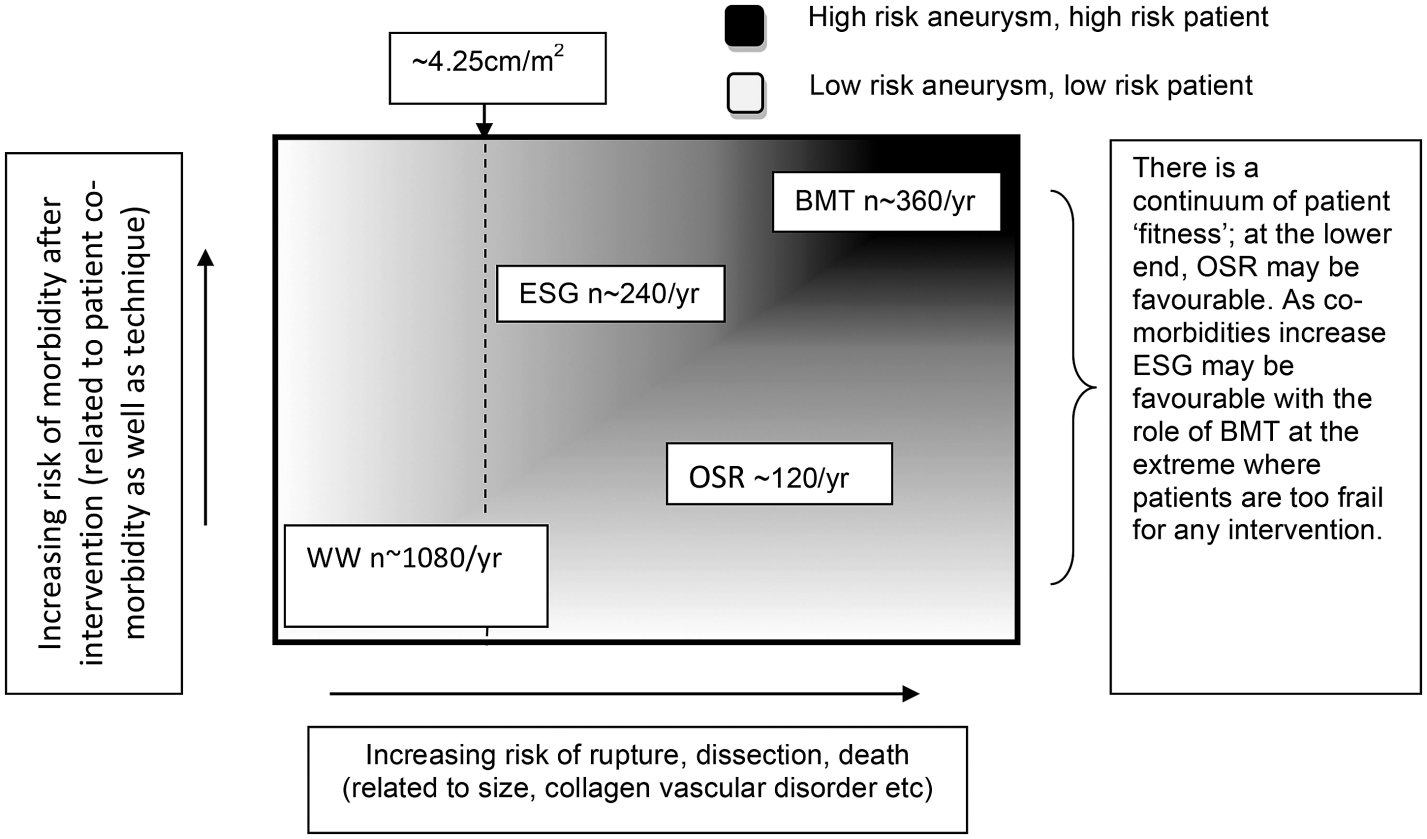
The expected UK populations (BMT, best medical therapy; ESG, endovascular stent grafting; OSR, open surgical repair; WW, watchful waiting).

Survey responses from the clinicians in this collaboration demonstrated that there is significant subjectivity in this decision-making process. At present, ESG tends to be performed for older patients with more comorbidity, since it is a less invasive intervention. OSR tends to be preferred for younger patients with less comorbidity, since they are more able to withstand the operation and it is felt that OSR may reduce the chance of further interventions in the future. On the basis of current practice, however, there are many patients who are considered equally appropriate for ESG and OSR based on patient and aneurysm factors (figure 2). Indeed, some of these patients may instead choose BMT. In this study, we will compare the clinical effectiveness and cost-effectiveness, as well as quality of life (QoL) outcomes of BMT, ESG and OSR among those patients thought to be suitable for more than one treatment.

### Existing literature

OSR has been the mainstay of treatment for CTAA for the past four decades. It is a complex operation requiring a large incision, but has been demonstrated to reduce mortality and can be performed reproducibly in cardiac surgical centres. Techniques have improved, but OSR still carries a risk of approximately 5% mortality and approximately 10% paraplegia. ESG for CTAA is a newer (approximately 10–15 years old) and less invasive technique. It has been shown to be technically feasible, and some studies have even demonstrated aneurysm shrinkage. It is, however, most often selected for patients who have been turned down for OSR. It cannot be performed in all patients due to specific technical requirements of the procedure. It is also relatively resource intensive as it requires a hybrid theatre and an appropriate theatre team.

Table 2 summarises the largest, most recent and longest running studies that compare clinical and cost outcomes for ESG and OSR.^3–8^ These were chosen as representative of the wider literature in the course of an informal literature review performed by the authors, searching PubMed, MEDLINE and the Cochrane database, using terms including (but not exclusively) ‘thoracic aneurysm’, ‘clinical outcomes’ and ‘cost-effectiveness’. All the studies acknowledge differences between surgical and endovascular cohorts which may influence the results that are reported. Stent grafting tends to be chosen for older patients with more comorbidity, many of whom were denied surgical repair. Despite its use on older, sicker patients, the risk of death, paraplegia or other complications appears to be less for ESG than for OSR. The need for reintervention, however, appears to be higher after ESG due to technical failures of the stent that accrue over time (16% at 4 years,^9^ 23% at 5 years, 37% at 8 years^10^), and with each reintervention there is an added risk of complication either due to the increased complexity of the procedure or deteriorating health of the patient.

There are few studies comparing the cost-effectiveness of ESG against OSR in the context of CTAA. These papers are also summarised in table 2. All the studies that appear in table 2 consider only in-hospital cost, thereby excluding the cost of reintervention. No formal economic evaluation has been performed. Therefore, there is a lack of economic data to guide decision-makers in the choices they must make in allocating the scarce resources available. A systematic literature review will be performed and included as part of the final report of this study.

**Table 2:** Summarised literature review

Study description	Key results		Comments
Desai *et al*, JTCVS 2012^3^ Retrospective cohort study *106 ESG vs 45 OSR* *10-year follow-up*	In-hospital mortality	ESG 2.6%, OSR 6.7% open; p=0.1	In multivariate analysis, age, COPD, diabetes and renal failure were predictors of late mortality but technique (ie, ESG vs OSR) was not
	Paralysis/paraparesis	ESG 3.9%, OSR 7.1%; p=0.2	
	10-year survival	Similar between groups (p=0.5)	
	Freedom from reintervention	ESG 85% at 10 years, OSR 0% at 10 years, p=0.2	
Gopaldas *et al*, JTCVS 2012^4^ Retrospective cohort study *2563 ESG* vs *9106 OSR* *2-year follow-up*	All-cause complications	ESG 22.9%, OSR 37.6%, p<0.001	The patients who had undergone ESG were older (mean age 69.5 vs 60.2, p<0.001) and had higher Deyo comorbidity scores The unadjusted cost for patients with ESG appeared to be lower, but did not prove to be significantly so on multivariate analysis The cost analysis only accounts for in-hospital cost for the index procedure
	Length of stay	ESG 7.7 days, OSR 8.8 days, p=1	
	In-hospital mortality	ESG 2.3%, OSR 2.3%, p=1	
	Average hospital charges	ESG US$46 636, OSR US$48 974, p<0.05	
Orandi *et al*, 2009^5^ Retrospective cohort study *763 OSR patients vs 267 ESG patients*	In-hospital mortality	ESG 7.7%, OSR 6.4% (p=.49)	The authors state that in-hospital costs were the same for patients with ESG and OSR (data not published for both groups), but this is assessed only in a subgroup of patients with no complications
	All-cause complications	ESG 20.4%, OSR 33.1% (p<0.001)	
	Mean length of stay	ESG 5 days, OSR 7 days, p=0.0015	
Bavaria *et al*, 2007^6^ Multicentre retrospective comparative trial *17 participating sites* *94 OSR patients vs 137 ESG patients.* *2*-*year follow-up (25.8 months (ESG) and 24.9 months (OSR))*	Perioperative mortality	ESG 2.1% vs OSR 11.7% p<0.001	
	In-hospital morbidity	ESG group had a significantly lower incidence of respiratory failure (4% vs 20%, p<0.001), renal failure (1% vs 13%, p=0.01) and paraplegia/paraparesis (3% vs 14%, p=0.003) The ESG group had a significantly higher incidence of peripheral vascular complications (14% vs 4%, p=0.015)	
	Mean ICU length of stay	ESG 2.6 days vs OSR 5.2 days, p<0.001	
	Mean hospital length of stay	ESG 7.4 days vs OSR 14.4 days, p<0.001	
	Estimated 2-year survival	78% ESG vs 76% OSR	
	Reinterventions	3 reinterventions in the ESG group within 2 years. None in the OSR group	
Dick *et al*, ATS 2008^7^ Retrospective cohort study *52 ESG* vs *70 OSR patients* *Mean follow-up of 34 months*	Perioperative mortality	ESG 8%, OSR 9%, p=0.25	Significant proportions (14–20%) of interventions were performed for acute rupture/dissection, ie, non-elective Also demonstrated lower incidence of pneumonia in the ESG group Does not capture preintervention QoL or demonstrate the expected early difference in QoL between ESG and OSR
	Hospital length of stay	ESG 11.6 days, OSR 18.3 days, p<0.001	
	*QoL score at 3 years (measured by SF-36 and hospital anxiety and depression scores)*	SF-36: ESG 83, OSR 93, p=0.66. Anxiety score: ESG 5, OSR 4, p=0.79. Depression score: ESG5, OSR 3.4, p=0.09	
Narayan *et al*, EJCTS 2011^8^ Retrospective cohort study *49 ESG (45% of these were for aneurysm)* vs *35 OSR (53% of these were for aneurysm)*	Total cost	£16 694 ESG vs £15 045 OSR p=0.41	Small study of within-hospital NHS costs only. Lack of detail regarding costing and operative indications. No long-term cost analysis for the lifetime of the patient. No wider costs including personal social services. No preference-based quality of life estimate

COPD, chronic obstructive pulmonary disease; ESG, endovascular stent grafting; ICU, intensive care unit; NHS, National Health Service; OSR, open surgical repair; QoL, quality of life.

### Why is this research needed now?

In the past 5 years, data have been published (mainly from US cohorts) regarding the clinical outcomes of ESG at a decade of follow-up. It has been shown to be clinically effective for some patients, but with an acknowledged complication rate of 10–15% (refs 3 and 4). Currently, there is no consensus on the best management strategy and timing of different interventions, and no UK-specific economic studies that assess outcomes beyond the chosen procedure. With this in mind, there is a need to generate further evidence regarding the cost-effectiveness of ESG, OSR and indeed BMT. This evidence is not currently adequate, but is urgently needed in the context of greater demand for treatment (an ageing population with a rising prevalence of CTAA) and limited National Health Service (NHS) resources.

## Methods and analysis

### Design

This is a prospective, multicentre, observational study with statistical and economic modelling of patients with a chronic aneurysm of the thoracic aortic arch (TAA) or descending thoracic aorta (DTA). Both these conditions are a subset of CTAA. The ascending aorta may also become aneurysmal, but is not considered in this study as ESG is rarely undertaken for this condition.

The adult aorta is expected to measure 2.39–2.98 cm^11^ at the point of the mid-DTA. Therefore, as a rough guide, aneurysm of the TAA or DTA is generally diagnosed at 4 cm.

### Patient enrolment

Each multidisciplinary team (MDT) in this collaboration will send information leaflets about the ETTAA study to general practitioners, cardiologists, cardiothoracic and vascular surgeons in their catchment areas, advertising the study and inviting referrals of all patients with CTAA ≥4 cm. Within the MDT forum, the clinical group will identify those patients who are eligible for the study. These patients will be approached by the investigating team local to them, led by the local principal investigator (PI) and assisted by a research nurse. Patients who give their consent to participate will be enrolled in the study by either the local PI or the local research team. By screening consecutive patients referred to the MDTs, rather than screening patients referred only to single specialists, we hope to minimise referral bias. We appreciate that we may miss some patients who are never referred to the MDT, but we hope that sending information leaflets and raising awareness of aortic MDTs within the primary care community will help support referral into the MDT system. Current practice is that specialists in the MDT will recommend either WW (if the risk of rupture is low) or intervention in the form of BMT, ESG or OSR when the risk of rupture is moderate-high (table 3). We recognise that patients who initially elect for a conservative option may later change their opinion, and this will be captured as a crossover from BMT to ESG/OSR. Patients enrolled in the study will be observed from the time of referral, up to and beyond treatment (BMT, ESG or OSR) until the study concludes at 5 years (median follow-up for this cohort therefore being 3 years).

**Table 3:** Study groups

WW	Patients with an aneurysm considered to be at a low risk of rupture will be started on prophylactic therapy as per internationally accepted guidelines detailed below. However, they will remain under surveillance in the form of an annual CT scan/MRI and MDT review. These patients’ data will contribute to the natural history component of our study
BMT	Patients who are considered unsuitable for *elective* ESG/OSR, or who refuse ESG or OSR, will be assigned to best medical therapy. This will follow international guidelines^11^ which recommend control of hypertension (BP<140/90 or 130/80 mm Hg for patients with diabetes or renal impairment), lipid profile optimisation (target cholesterol <70 mg/dL), smoking cessation and other atherosclerotic risk-reduction measures to reduce the risk of stroke, MI, heart failure and cardiovascular death. We recognise that patients who initially elect for a conservative option may later change their opinion and this will be captured as a crossover from BMT to ESG/OSR
ESG	Endovascular repair of the aneurysm via transluminal introduction of a stent-graft under X-ray guidance. Hybrid procedures that comprise a combination of a conventional surgical component and a transluminal repair are also included in this group since the aim of such techniques is to minimise the overall invasive nature of repair
OSR	These patients will undergo replacement of the aneurysmal aorta with a prosthetic conduit via a sternotomy or thoracotomy with circulatory support

BMT, best medical therapy; BP, blood pressure; ESG, endovascular stent grafting; MDT, multidisciplinary team; MI, myocardial infarction; OSR, open surgical repair; WW, watchful waiting.

### Measurement of cost and outcomes

Primary study outcomes are:

- Aneurysm growth;
- QoL;
- Freedom from reintervention;
- Freedom from death or permanent neurological injury;
- Incremental cost per quality-adjusted life year (QALY) gained.

These in turn will be derived from a fuller list of parameters (table 4). These parameters have been chosen by the collaborators on the basis of current literature and previous risk modelling. As described below, primary outcomes will be compared in patients thought to be suitable for more than one treatment. In non-comparable patients, the primary outcomes will be described.

**Table 4:** data to be collected from study patients

Patient factors	Aneurysm factors	Outcomes
Gender	Connective tissue disorder	*Technical outcomes*
Age	Presenting symptoms of aneurysm	Treatment (BMT/ESG/OSR)
Height	Extent of aneurysm	Reoperation for bleeding
Weight	Aortic diameter immediately proximal to aneurysm	Access vessel injury
Hypertension	Aortic diameter immediately distal to aneurysm	Endoleak
Diabetes mellitus	Maximum diameter of thoracic aorta on presentation	Endoleak treatment
Smoking history	Aneurysm length	Conversion to open surgery
LV function	Proximal neck length	Infection
Coronary artery disease	Distal neck length	Fistulae
Valvular heart disease		Reintervention
COPD		Aneurysm growth rate
Creatinine		*Clinical outcomes*
Previous neurovascular injury		Death
Extracardiac arteriopathy		CVA (neuro deficit >48 h)
Logistic Euroscore		Myocardial infarction
Previous cardiac/aortic intervention		Mechanical respiratory support >48 h
Family history of aneurysm		Renal replacement therapy
Living status		Paraplegia
EQ-5D-5L		DVT/PE
		EQ-5D-5L
		*Cost outcomes*
		Operating room time
		Hybrid theatre time
		Prosthesis
		Blood products
		ICU days
		HDU days
		Ward days (preoperative+postoperative)
		Medications
		Investigations in-hospital
		Outpatient visits
		Outpatient investigations
		Treatment of complications
		Primary care visits

BMT, best medical therapy; COPD, chronic obstructive pulmonary disease; CVA, cerebrovascular accident; DVT, deep vein thrombosis; ESG, endovascular stent grafting; HDU, high-dependency unit; ICU, intensive care unit; LV, left ventricle; OSR, open surgical repair; PE, pulmonary embolism.

All data collection will be prospective, performed by the research team local to the patient, either in person during a hospital/clinical attendance or over the telephone. Procedure-related complications/clinical outcomes will be collated from medical records, and QoL will be determined by patient-completed EQ-5D-5L questionnaires. Clinical outcomes and EQ-5D-5L scores will be recorded at initial review (time zero), 3, 6, 12, 18 and 24 months, and then annually until the follow-up concludes at 5 years. If a patient undergoes ESG or OSR, the ‘clock’ will be reset on the day of intervention. Clinical outcomes and QoL data will then be collected preprocedure, at discharge, 3, 6, 12, 18 and 24 months, and then annually after intervention until the follow-up concludes. This will allow a comparison of QoL in the early period after ESG or OSR since there is likely to be a significant difference in this phase.

In contrast, technical outcomes and aneurysm size will be assessed less frequently since they require interval CT scan or MRI. These will be performed in line with current practice guidelines (minimum once per year, with further scans performed as clinically indicated) and supported by morphological analysis in the Corelab to reduce reporting bias. The Corelab will be run by St Georges Hospital and led by Professor Matt Thompson. It is a facility where anonymised scans will be analysed and reported by independent experts to ensure consistent and uniform reporting to defined standards. If discrepancies are found in excess of the expected interobserver error, then the provenance of the scans will be traced back and investigated. In addition to validating aneurysm growth rates, the Corelab will also provide an independent analysis of technical outcomes following intervention.

The primary outcomes will be reported at the completion of the study, with an anticipated median follow-up of 3 years.

### Comparison of treatment groups for clinical effectiveness

On the basis of initial audits of the participating centres, there is substantial heterogeneity among clinicians in the timing and/or nature of interventions, with some centres favouring ESG and others focused on OSR. This will create a substantial overlap in characteristics between the populations of patients undergoing ESG and OSR. Therefore, it will be possible to compare treatment groups for clinical and cost-effectiveness of these two groups based on the actual treatment undertaken. This will require methods specifically designed to address bias inherent in observational studies, and these are described in more detail in the statistical analysis section below.

The overlap between populations assigned to medical therapy and the intervention groups is less clear. *If* there is substantial heterogeneity among clinicians in the patient characteristics of this group, then clinical and cost-effectiveness comparisons can be undertaken using methods similar to the ESG-OSR comparison above. On the other hand, *if* all clinicians intervene with either ESG or OSR at a similar stage in aneurysm development, and for similar patients, then WW and BMT patients will form two distinct groups, in terms of aneurysm morphology, and the defined population and their outcomes will be reported. No comparisons will be undertaken.

### Comparison of treatment groups for cost-effectiveness

This will involve both a ‘within’ study patient-level analysis and a model-based analysis to extrapolate outcomes into the longer term. The within-study analysis will take the form of a cost-utility analysis with outcomes reported in terms of incremental cost per QALY gained, and also costs and QALYs for each intervention compared. The methods to conduct this analysis are explained in the economic analysis section below but will need to address the same issues and hence use methods similar to those required to assess clinical effectiveness from an observational study as noted in (3) above. The base case analysis will compare similar groups of patients where each group initially receives one of the health technologies under investigation. The long-term analysis will be based on a Markov model. The model structure will describe the sequence of events from the point where an individual receives one of the interventions under investigation. It will include perioperative and postoperative complications for surgical interventions and side effects of medical treatments. It will also describe the potential sequence of events that might occur over time. The development of the structure of the model will be informed through consultation with the study team and using information from the literature. The methods used to populate the model and analyse the data are described in the economic analysis section below.

### Inclusion/exclusion criteria

*Inclusion* *criteria*: All patients who are over 18 years of age with chronic aneurysm of the arch or DTA and who can provide valid written consent are eligible for the study.

*Exclusion criteria*: Patients with acute dissection or malperfusion syndromes (such as myocardial infarction, acute stroke or limb ischaemia) will be excluded. This is because the clinical picture signifies a type B acute aortic syndrome, and the role of surgery and stenting in this context has been investigated by others.

### Data collection

The data to be collected is shown in table 4. Data will be collected from a variety of sources, but mainly medical records and patient feedback questionnaires for clinical events and QoL; CT scan/MRI for aneurysm morphological data; unit costs of services used will be obtained from parallel costing exercises and use study-specific estimates, NHS reference costs, manufacturer/supplier costs and other publicly available data.

### Ethics and dissemination

The study will culminate in a Health Technology Assessment monograph describing the study and its results in detail, which will allow us to make recommendations for practice and policy in the UK. We anticipate a number of publications describing:

- Changes in aneurysm size over time;
- Selection criteria for ESG, OSR and BMT;
- Factors affecting outcomes after ESG and OSR;
- Comparative clinical outcomes after ESG and OSR in those patients who were eligible for both treatments;
- QoL and cost-effectiveness in patients for whom more than one treatment is appropriate;
- Geographical variation in patterns of disease.

Furthermore, we will provide an analysis of patient-specific and aneurysm-specific factors (if there are any) which predict good or poor outcome. It is hoped that identification of such factors will allow us to propose a draft guideline for the indications for BMT, ESG or OSR in patients with CTAA. We also anticipate that there may be a number of additional publications:

- Geographical variance in patterns of disease, QoL and patient choices;
- Influence of waiting lists for ESG versus OSR on QoL;
- Statistical methodology for estimation of aneurysm growth from multiple related but different evidence sources;
- Methods for robust cost-effectiveness comparisons in the absence of clinical trial data.

Papers regarding current and future decision-making will arise from our planned Delphi exercise.^12^ The process will be in two stages, each comprising two rounds. The first stage of the exercise will be conducted at the beginning of the study and is aimed at charting existing practice and the prevailing concepts underpinning that practice. The second stage is conducted when results of the observational study are available for dissemination. The aim of this stage is to develop consensus regarding the most appropriate method of managing CTAA in the UK. Participants will be drawn to represent as wholly as possible the existing service provision with no regard to perceived or established treatment or referral biases since that gives the best potential to realise the aims of both stages of the exercise. The exercise will be conducted using RAND methodology.^12^ The first rounds of both stages will be conducted electronically with an estimated total of 108 case vignettes representing combinations of different morphological, age, fitness and connective tissue factors. The second rounds will require fewer case vignettes and will be conducted as workshops in conjunction with a national conference.

The findings from this study will be reported locally, nationally and internationally in the form of presentations and papers to medical professionals as well as patient groups. This information will also be available via patient information leaflets and the study website which will be designed with the help of our patient representatives.
